# Homeostasis and Dysbiosis of the Intestinal Microbiota: Comparing Hallmarks of a Healthy State with Changes in Inflammatory Bowel Disease

**DOI:** 10.3390/microorganisms10122405

**Published:** 2022-12-05

**Authors:** Jasminka Talapko, Aleksandar Včev, Tomislav Meštrović, Emina Pustijanac, Melita Jukić, Ivana Škrlec

**Affiliations:** 1 Faculty of Dental Medicine and Health, Josip Juraj Strossmayer University of Osijek, Crkvena 21, 31000 Osijek, Croatia; 2 University Centre Varaždin, University North, 42000 Varaždin, Croatia; 3 Institute for Health Metrics and Evaluation and the Department of Health Metrics Sciences, University of Washington, Seattle, WA 98195, USA; 4 Faculty of Natural Sciences, Juraj Dobrila University of Pula, 52100 Pula, Croatia; 5 General Hospital Vukovar, Županijska 35, 32000 Vukovar, Croatia

**Keywords:** microbiota, microbiome, dysbiosis, inflammatory bowel disease, Crohn’s disease, ulcerative colitis, nutrition

## Abstract

The gut microbiota, which represent a community of different microorganisms in the human intestinal tract, are crucial to preserving human health by participating in various physiological functions and acting as a metabolic organ. In physiological conditions, microbiota–host partnership exerts homeostatic stability; however, changes in intestinal microbiota composition (dysbiosis) are an important factor in the pathogenesis of inflammatory bowel disease and its two main disease entities: ulcerative colitis and Crohn’s disease. The incidence and prevalence of these inflammatory conditions have increased rapidly in the last decade, becoming a significant problem for the healthcare system and a true challenge in finding novel therapeutic solutions. The issue is that, despite numerous studies, the etiopathogenesis of inflammatory bowel disease is not completely clear. Based on current knowledge, chronic intestinal inflammation occurs due to altered intestinal microbiota and environmental factors, as well as a complex interplay between the genetic predisposition of the host and an inappropriate innate and acquired immune response. It is important to note that the development of biological and immunomodulatory therapy has led to significant progress in treating inflammatory bowel disease. Certain lifestyle changes and novel approaches—including fecal microbiota transplantation and nutritional supplementation with probiotics, prebiotics, and synbiotics—have offered solutions for dysbiosis management and paved the way towards restoring a healthy microbiome, with only minimal long-term unfavorable effects.

## 1. Introduction

The human gastrointestinal tract (GIT) is a home to an abundant and protean congregation of more than 100 trillion microorganisms that live in peaceful coexistence with their hosts [1]. This complex but well-organized community is known as intestinal microbiota, microflora, or normal gut flora [2]. During the last decade, novel and emerging technologies have enabled phylogenetical identification and quantification of the gut microbiota constituents, primarily by relying on the analysis of nucleic acids extracted from stools [3,4]. The term for the collective genetic content of this living microbiota is known as a microbiome [5]. Although these terms are often used interchangeably, this difference should be acknowledged for much better understanding of studies in the field.

The availability of whole-genome sequencing, in conjunction with metagenomic/metabolomic techniques, have opened the door to appraising the composition of gut microflora in different contexts. As a result, it has become possible to pinpoint differences in healthy and diseased states [6,7]. However, it quickly became evident that due to various external and internal factors (most notably genetics, diet, and the environment), it is impossible to generalize a “healthy” gut microbiome on a population level [8,9,10,11]. Nevertheless, specific combinations of microbial species have been repeatedly linked to certain conditions, diseases, or geographical regions. Furthermore, dysbiosis, or disturbed balance of microbiota, is a hallmark of many different diseases—most notably inflammatory bowel disease [12,13].

In this review, we aimed to define the role and composition of normal gut microbiota and its importance for maintaining intestinal homeostasis, but also to highlight the differences of microbial communities observed between and within individuals. We also emphasized intestinal dysbiosis and its links to enteric and other diseases, with an emphasis on inflammatory bowel disease. Manifold ways of manipulating gut microbiota have entered quotidian clinical practice and enabled the reinstitution of a lost balance of gut bacteria, which is a very pertinent discussion as well. The ultimate goal of a large body of research on human microbiota is translational in nature and aimed at answering the most important question: how to optimize disease management and restore a healthy state.

## 2. The Diversity of Gut Microbiota

The intestinal tract is one of the most colonized known habitats for microorganisms, and this is particularly true for the colon, which harbors between 10^11^ and 10^12^ bacterial cells per milliliter [14,15]. Since the totality of gut microbiota and its symbiotic relationship with the host organism are characterized not only by substantial diversity but also profound resilience and stability, such host–microbiota interrelation can be viewed in terms of a “superorganism” performing various metabolic and immune functions [16,17].

### 2.1. The Role and Composition of Gut Microbiota

Intestinal bacteria play a significant role as principal regulators of digestion; specifically, microbial residents of the gastrointestinal tract have a role in extracting, synthesizing, and absorbing many metabolites and nutrients (such as amino acids, lipids, bile acids, vitamins, and short-chain fatty acids) [17]. Furthermore, they are responsible for antagonistic microbial interactions, which means that their mere presence (alongside the production of biocins and the utilization of available nutrients) prevents potentially pathogenic bacteria from colonizing this niche, but also preserves the integrity of the gut epithelium [17,18,19]. Other competition mechanisms include the secretion of antimicrobial peptides [20], pH modifications [21], the control of innate and adaptive immune cells [22], and influencing cell signaling pathways [23].

The native gut microbiota can be recognized very early in life (i.e., between 4 and 36 months of age), but its relative stability can be seen upon reaching the age of two [17]. The constituents of gut microbiota are several types of microorganisms, which include bacteria, fungi, viruses, protists, and even archaea; however, most studies have mainly concentrated on bacteria. Additionally, although more than 160 bacterial species have been described to date, they belong to only a handful of bacterial phyla [17]. Of those, the dominant ones are phyla Bacteroidetes, Firmicutes (or Bacillota), Proteobacteria, Actinobacteria, Verrucomicrobia, and Fusobacteria, with 90% of intestinal microbiota represented by Bacteroidetes and Firmicutes [24,25]. The most dominant genera within the latter phylum (Firmicutes) are *Clostridium*, *Lactobacillus*, *Bacillus*, *Enterococcus*, and *Ruminicoccus* [17,25]. Conversely, the *Bifidobacterium* genus is well known but belongs to the less abundant Actinobacteria phylum [24]. Moreover, fungal genera which are found in the gut include yeasts such as *Candida* and *Saccharomyces*, as well as molds such as *Aspergillus*, *Rhodotorula*, and *Penicillium*, among others [26,27]. In addition, the human gut virome harbors eukaryotic viruses (infecting human cells in the gut) and prokaryotic viruses (primarily infecting bacteria), of which the latter predominate (i.e., more than 90%) [28]. Further studies are needed to fully elucidate archaeal and parasitic constituents of human gut microbiota.

Different studies have tried to define a core microbiota in the human intestine with the use of longitudinal analysis and comparisons of fecal 16S ribosomal RNA (rRNA) [29]. A stable bacterial core was represented by the genera *Bacteroides*, *Faecalibacterium*, *Eubacterium*, *Ruminococcus*, *Alistipes*, *Roseburia*, *Clostridium*, and *Blautia*; moreover, *Faecalibacterium prausnitzii*, *Ruminococcus obeum*, and *Oscillospira guillermondii* were the top three taxa shared by all adults in many studies [21,29]. This is important to understand, as this can provide additional insight into bacterial metabolites that can impact host processes and metabolic capabilities. Likewise, the characterization of intestinal microbes that are able to produce vitamins belonging to the B complex via coordinated bacterial cross-feeding is increasingly important in our understanding of a sundry of their metabolic functions [29].

### 2.2. The Variations of Gut Microbiota

Although the functions of the intestinal microbiota are highly conserved between different individuals, a specific combination of microbial genera and species is found within each person’s gut as a result of inter- and intra-individual variability during a lifespan [17,30]. Hence, a specific hallmark of every individual is a particular cluster of bacteria, which can be subsequently grouped into different enterotypes [24,25]. More specifically, there are three enterotypes distinguished by dominant clusters of one of three bacterial taxa: enterotype I, characterized by *Bacteroides*; enterotype II, characterized by *Prevotella*; and enterotype III, characterized by *Ruminococcus* [17,30]. It has to be noted that they do not merely represent a systematic tally of bacterial species but also convey a true functional and well-balanced association. Additionally, even though enterotypes cannot be considered permanent for any individual, they are indeed stable, highly characteristic, defined by nutritional habits, and may be restored in instances when they are modified [17,25]. By encompassing specific clusters of bacteria with their functional traits, each enterotype has a specific pathway for utilizing fermentable substrates in the colon to generate energy. For example, energy in the enterotype I cluster is derived from carbohydrates using pentose phosphate and glycolysis pathways. In contrast, enterotypes II and III are known for degrading mucin glycoproteins found in the mucosal layer [17].

Gut microbiota can also show variations in accordance with the anatomical regions of the intestines due to differences in substrate availability, physiological processes, pH levels, oxygen tension, flow rates of digested food, and secretion content from the host [31]. For example, the small intestine is a rather unfavorable milieu for microbial colonizers due to high bile concentrations and rapid transit time. Conversely, neutral/gently acidic pH and protracted flow rates shape a suitable environment to harbor the largest community of microorganisms [31]. Many studies imply how the gut microbiome can be affected by a myriad of host characteristics, including geographical region, ethnicity, and socioeconomic status [30,32,33,34]. There is also increasing evidence based on the seasonality of gut microbiota that has to be considered, as such shifts in microbiota composition can affect the seasonal pattern of infectious and non-infectious disease incidence and recurrence [35]. Likewise, shifts in the intestinal microbiota composition can also be influenced by physical activity and exercise [36]; for example, studies have found that the genus *Veillonella* is enriched within the intestinal flora of athletes [37].

## 3. Maintaining Homeostasis and Gastrointestinal Health

Mammals and their commensal microorganisms have co-evolved towards mutualism and homeostasis. Consequently, the microbiota–host partnership is crucial for maintaining a healthy state, but also for influencing susceptibility to disease development [38]. In short, homeostasis requires a healthy gut microbiota. In the homeostatic stage, the microbiota is requisite for the host’s metabolic functions, the development of the immune system, and resistance to exogenous pathogens. It strongly influences short- and long-term homeostasis [39,40,41]; however, as already mentioned, the GIT microbiota varies over time and differs among healthy individuals [17,30,42].

### 3.1. What Influences Homeostasis?

Homeostatic stability is greatly influenced by environmental factors, as well as host genetics [41]. Thus, salient environmental factors such as diet can undoubtedly influence the homeostatic configuration of the GIT microbial ecosystem. The most common ingredients of the human diet are carbohydrates, proteins, and fats; consequently, a change in their ratio can lead to changes in microbial diversity in the GIT. Other factors, such as antibiotic usage, lifestyle, immunodeficiency, age, and gender, certainly have an impact as well [40,42,43,44,45,46,47,48,49,50,51].

Regarding microbial ecology, factors affecting microbial growth, metabolic functions, colonization efficiency, and communication between microbial species significantly influence the state of homeostasis [52]. Although environmental factors are the main determinants of GIT microbiota diversity and may have an impact on homeostasis, host genetic variation should not be discounted either. Finally, genetic predisposition to diseases can depend on the microbiome [53,54]. More specifically, as stated previously, a healthy microbiome depends on an assemblage of microbial species that can carry out specific sets of biomolecular functions [38].

### 3.2. What Is Affected by Homeostasis?

Microbiome research has revealed that the gut microbiome actively influences multiple host functions, including circadian rhythmicity, nutritional responses, metabolism, and immunity [55,56,57].

In fact, host–microbiota interactions on the intestinal mucosa are the best-studied interactions, as the intestinal mucosa represents the largest surface area in contact with the antigens from the external environment. The immune system plays a vital role in gut homeostasis as the gut microbiota maintains a symbiotic relationship with the gut mucosa [39,58]. A special ability of the intestinal immune system is immune tolerance against a large and changing number of harmless microorganisms. In contrast, immune responses against pathogenic infections and commensal intrusions into sterile parts of the body are preserved [59]. During homeostasis, the host’s immune response to the intestinal microbiota is strictly compartmentalized to the mucosal surface. A dense mucus layer separates the intestinal epithelium cells from the microbiome [60]. Intestinal IgA and microbiota have a regulated mutualistic relationship, where a diverse and selected IgA repertoire maintains a well-balanced and diverse microbiome [61].

Emerging evidence has underscored specific crosstalk between the GIT microbiota and extra-intestinal organ immunity; more specifically, microbiome-associated metabolites translocate from the intestinal lumen to various organs such as the brain, lung, and liver through the circulatory system, and thus induce tissue-specific local immune responses [62,63,64,65].

In order to affirm the importance of homeostasis, it is necessary to emphasize the functions of the normal gut microbiota, such as the metabolism of nutrients (digestion of carbohydrates, proteins, and lipids), the synthesis of vitamin K, the breakdown of various polyphenols, the metabolism of xenobiotics and drugs, antimicrobial protection, immunomodulation, and the preservation of the integrity of the GIT barrier and structure [39,66,67,68].

### 3.3. Resistance and Resilience

From a microbial ecology perspective, a healthy microbiome is resistant to stress and perturbation and has the propensity to recover to a healthy functional profile. Therefore, it has a certain degree of resilience to external (change in diet, entry of pathogens, medication) or internal changes (age, immunodeficiency) [38,40,69,70]. Thus, either perturbation can be counteracted, or the microbiome can shift from a healthy state. After that, the resilient microbiome can return to a healthy state, which may or may not be the original state, or it can transition to an unhealthy state. From an ecological point of view, factors that assist resilience and resist perturbation could promote human health [41,71]. Although certain members of the bacterial community may play an important functional role in the field of resistance to infection, the ability to occupy host niches and generate effective resistance to pathogens may depend on the unique functional activity of a specific microorganism [69,71].

Considering all of the above, it is challenging to define microbial health since it does not encompass a static state, but rather a dynamic balance [40,69].

## 4. Intestinal Dysbiosis and Enteric Diseases

Bacterial species found in the digestive tract, through direct contact with host cells or indirect communication through bacterial metabolites, can affect the maintenance of homeostasis and trigger inflammatory mechanisms [72]. An intact intestinal epithelium represents the first line of defense against pathogenic and commensal bacteria invasion with complex mucosal and solid intercellular junctions [73]. In situations where the intestinal barrier is damaged, there is a possibility for bacteria and their toxins to translocate by using a paracellular route through damaged tight intercellular junctions, or a transcellular route directly through cells into the portal circulation [74].

**Figure 1:**
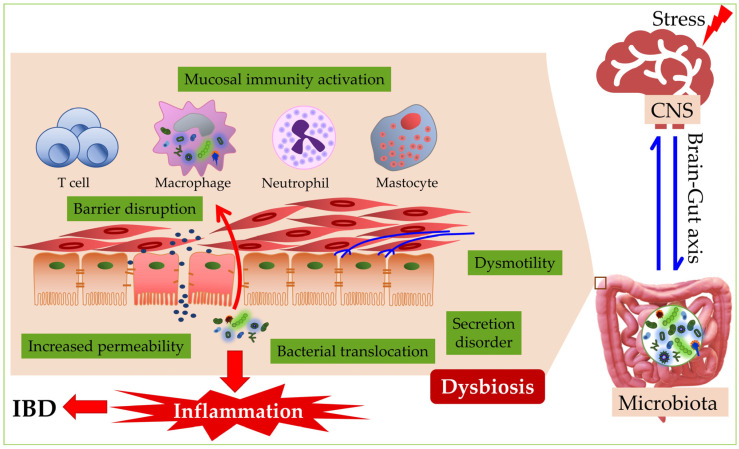
The influence of stress on inflammatory bowel disease. Stress promotes the activation of the brain–gut axis, contributing to the development of inflammatory bowel disease (IBD) through dysbiosis, changes in secretion and mobility, disturbance of the intestinal barrier, and the release of inflammatory mediators. CNS—central nervous system.

The integrity of the intestinal barrier can be disrupted by dysbiosis, which worsens a whole series of intestinal defense functions, and creates a predisposition to the onset of various diseases. For this reason, the microbiota and the integrity of the intestinal barrier are necessary to preserve the health of the digestive system [75]. Dysbiosis occurs when the balance of intestinal microbiota is disturbed and when potentially pathogenic microorganisms predominate at the expense of commensal bacteria, based on which a metabolic or immune response of the host may occur [76] (Figure 1). It has been observed that, in people with immune-mediated diseases, there are differences in the microbial communities compared to healthy individuals, which suggests that the pathogenesis stems from the disruption of the structure of the commensal bacterial community, subsequently leading to the development of immune-mediated diseases [77]. Three types of dysbiosis are known (Table 1), and in most instances, they occur simultaneously.

Various environmental factors often trigger dysbiosis, so broad-spectrum antibiotics used to treat infections significantly impact the microbiota [79]. In addition, some antibiotics have long-lasting effects on the microbiota, which lead to the permanent loss of certain microorganisms; specifically, the overgrowth and persistence of pathogenic microorganisms [80]. Environmental factors include nutrition, stress, and various infections [81], which highlights their pivotal role in the complex pathogenesis of inflammatory bowel disease, but also other important conditions (Table 2) [78].

The pathogenic involvement of the host’s microbiota in inflammatory bowel diseases represents a two-way relationship between altered immune function (mucosal barrier, immune regulation) and modified bacterial community (its features, functions, and metabolites) [82]. Pathogenesis is triggered by metabolic signaling from the intestinal microbiota, which carries a frank potential to influence the host, i.e., its health status [81]. Microbial signaling occurs through the structural components of bacteria or metabolites, and is transmitted through the intestinal epithelium, which is responsible for communicating with distant organs [83]. Upon conveying these signals, their influence on the organs is enabled through subsequent signaling with the help of hormones or nerves [84]. The association between inflammatory bowel diseases and gut microbiota has been revealed by 16S rRNA sequencing analysis [85].

In dysbiosis, the increased number of organisms known as pathobionts can modulate the expression and activation of Toll-Like Receptors (TLRs), which can then lead to a pro-inflammatory response in the gut and locations outside the intestines [86]. In contrast, NOD-Like Receptors (NLRs) have beneficial or detrimental effects that rely on antimicrobial factors and the pro-inflammatory cytokine profile upon activation of the intestinal microbiota [87].

In patients with inflammatory bowel disease (IBD), there is a decrease in the number of bacteria from the phylum Firmicutes, which includes Gram-positive bacteria with rigid or semi-rigid cell walls that are predominantly from the genera *Bacillus*, *Clostridium*, *Enterococcus*, and *Lactobacillus Ruminicoccus*, and an increase in Bacteroidetes, which includes approximately 7000 different types of Gram-negative bacteria that are predominantly from the genera *Alistipes*, *Bacteroides*, *Parabacteroides* and *Prevotella* [88] (Table 3). In addition, there is a significant increase in the number of bacteria from the family *Enterobacteriaceae*, which includes *Escherichia coli*, *Klebsiella* spp., and *Shigella* spp. [89]. In the digestive system of patients with IBD, there are an increased number of bacteria belonging to the genera *Streptococcus*, *Lactobacillus*, and *Enterococcus*, which can produce significant amounts of hydrogen peroxide; this, in turn, leads to a decrease in the population of anaerobic bacteria and can stimulate the cells of the immune system to release pro-inflammatory cytokines, but also stimulate apoptosis of intestinal epithelial cells deprived of the protective mucus layer [90]. Furthermore, the *Enterococcus* genus contributes to the disruption of the intestinal barrier and inflammation of the intestine using a metalloproteinase that cleaves epithelial cadherins [91]. Likewise, a positive correlation between alkaline phosphatase levels was observed, indicating biliary pathology in connection with *Enterococcus* [92]. Additionally, patients with IBD showed increased biofilm production of *Enterococcus* strains compared to strains from the control group [93]. Based on recent research results, *Fusobacterium* is associated with the severity of intestinal inflammation [94]. In addition to bacterial dysbiosis, there is a significant role of fungal dysbiosis in IBD, resulting in a higher representation of the genus *Exophiala* and a reduced proportion of *Saccharomyces cerevisiae* species [95].

## 5. The Connection of Dysbiosis to Inflammatory Bowel Disease

The main conditions within the umbrella of IBD, Crohn’s disease (CD) and ulcerative colitis (UC), are chronic conditions of the gastrointestinal system characterized by alternating episodes of relapse and remission, which affect about 3 million people in the USA and Europe [96]. Interactions between environmental factors, dysregulated immune response, host genetics, and changes in intestinal microbiological composition are involved in the disease pathogenesis [97]. CD can affect any part of the digestive system, but most often, it is observed in the last part of the small intestine or the large intestine, while UC disease is exclusively related to the large intestine [98]. In addition, the incidence and prevalence of IBD are closely related to urban lifestyle and northern latitudes, while these conditions are relatively rare in Africa, Asia, and Latin America [99].

IBD can appear throughout the lifetime, from the first year of life to old age, with pronounced peaks of incidence between 15 and 30 and between 60 and 80 years [100]. In the last decade, a significant increase in incidence has been recorded worldwide [101,102]. Although the etiology and pathogenesis of IBD are thus far unclear, it is evident that dysbiosis within the intestinal microbiome represents a crucial factor in the development of IBD and an essential piece in the development of mucosal lesions [103]. The microbiota of patients with IBD significantly differs from those in healthy individuals and is characterized by a smaller abundance and diversity of microorganisms [104]. Both qualitative and quantitative changes are present in the composition and function of the microbiota associated with IBD [105].

The microbial population changes in the digestive system of patients with CD and UC. Bacteroidetes and Firmicutes, as well as *Clostridium leptum* and *Clostridium coccoides* groups, were shown to be reduced [106]. In several studies on the same group of patients, a reduced representation of *F. prausnitzii* (a member of the *C. leptum* group), characterized by anti-inflammatory properties, may increase the risk of postoperative recurrent ileal disease and lead to an increased abundance of *E. coli* [107].

The reduced prevalence of *F. prausnitzii* (Firmicute), which belongs to the butyrate-producing *Clostridia* cluster IV species, illuminates the reduced amount of short-chain fatty acids in the fecal samples of patients with IBD [108]. Butyrate, in addition to strengthening the mucosal barrier by inducing the production of antimicrobial peptides and mucins [109], represents the primary source of energy for the epithelial cells of the large intestine [110] and also serves as an inhibitor of the expression of pro-inflammatory cytokines in intestinal mucosa through a mechanism involving hyper-acetylation of histones and suppression of NF-κB signaling [111].

Ulcerative colitis and Crohn’s disease belong to the group of chronic inflammatory bowel diseases [112]. In people with active CD, as a sign of dysbiosis, there is a decrease in the number of *Firmicutes* bacteria belonging to the *Ruminococcaceae* and *Lachnospiraceae* families [113]. This is important because these are genera of bacteria that normally belong to the families of human intestine bacteria that produce butyrate [114]. In CD, an increase in the number of sulfate-reducing bacteria has been noted, during which hydrogen sulfate is formed, which damages the intestinal barrier and promotes inflammation. An example of such a bacterium is *Desulfovibrio* [115]. Additionally, the abundance of *Escherichia* and *Shigella* was significantly increased compared to healthy people [116]. The adherent-invasive *E. coli* (AIEC) group is involved considerably in CD development. The data show that it was isolated from the intestines of 6% of healthy people, and this percentage is significantly higher in CD and amounts to about 38% [117]. In addition, the number of mucolytic bacteria such as *Bifidobacterium bifidum*, *Bacteroides fragilis*, *Runinococcus gnavas*, and *Ruminococcus torques* is rising, which leads to the breakdown of protective mucus and increased bacterial invasion of the mucous membrane [118]. *Mycobacterium avium paratuberculosis* (MAP), which usually causes chronic granulomatous ileitis, is associated with CD; namely, a more frequent presence of antibodies and reactive T cells against MAP and a higher level of MAP-DNA in the mucosa of patients with CD compared to the control group [119].

The research results determined that the composition of bacterial species is substantially modified in people suffering from UC; more specifically, bacteria belonging to the phylum Firmicutes are of lower abundance, while the number of bacteria from the phylum Proteobacteria is high [106]. In addition, it is important to note that the number of bacteria that have a protective role in GIS is significantly decreased, including the genera *Bacteroides*, *Eubacterium*, and *Lactobacillus* [106]. The number of bacteria that produce short-chain fatty acids, such as *F. prausnitzii* and *Clostridium butyricum*, is decreased in patients presenting with UC, which can affect regulatory T cells, primarily their differentiation and expansion [120]. Individuals experiencing a relapse of UC have a higher proportion of Bacteroidetes, but a much lower proportion of *Clostridiales* [121]. Moreover, a correlation between butyrate-producing bacteria (such as the aforementioned *F. prausnitzii* and *Roseburia hominis*) and UC disease activity has repeatedly shown an inverse trend [122]. Several other studies demonstrated a link between active UC and lower abundance of *Akkermansia municiphila* and *Roseburia* spp. [123,124]. Finally, it has to be emphasized that the alterations in intestinal microbiota composition in patients with UC are also related to changes in microbial metabolism; for example, increases in sphingosine-1-phosphate and trimethylamine N-oxide are often observed [122].

In patients with CD, the fecal fungal community is also disturbed, accompanied by an increased diversity and prevalence of the fungi *Candida albicans*, *C. neoformans*, and *Aspergillus clavatus* [125]. In addition, norovirus infection is mentioned as a possible trigger for the development and relapse of CD disease [126]. In patients with UC, some changes have been observed in regard to bacteriophage populations; more specifically, a large number of DNA viruses have been observed in gut mucosa (such as *Caudovirales* bacteriophages), but there also a decrease in *Caudovirales* richness and diversity [122,127].

Significant differences were found in a study that studied the microbiota of pediatric patients with CD and compared it with a healthy control group. Specifically, CD patients had an increased abundance of *Enterobacteriaceae*, *Pasteurellaceae*, *Veillonellaceae*, and *Fusobacteriaceae* and a decreased amount of *Erysipelotrichales*, *Bacteroidales*, and *Clostridiales* compared to healthy controls [128]. Additionally, although dysbiosis is more pronounced in children with CD when compared to children with UC, there are also some characteristic hallmarks in pediatric patients with UC, such as a reduction in abundance of *Akkermansia* spp. and *Eubacterium rectale*, and the expansion of *E. coli* [129]. In addition, an absent response to steroids given to children with acute and severe forms of UC has been linked to a substantial reduction in bacterial diversity in comparison to steroid responders and healthy controls [130].

Hence, as a result of dysbiosis, various harmful products are produced that break down and thin the protective mucus layer and damage the epithelial layer [104]. Due to the impaired integrity of the epithelial barrier, microorganisms can penetrate the lamina propria, which results in an excessive immune response of the host [131]. This leads to a breakdown of immune tolerance to one’s intestinal microbiota while stimulating an inflammatory reaction, leading to tissue damage [132].

## 6. Manipulating the Gut Microbiota

**Figure 2:**
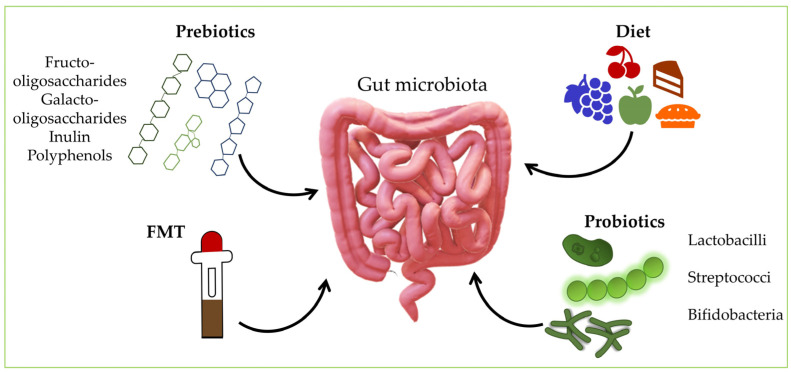
Schematic representation of different treatments of gut microbiota manipulation. FMT—fecal microbiome transplantation.

The intestinal microbiota is an essential component of who we are, and the mutual dialogue between the microbiota and the host results in lifelong epigenetic programming [133]. The significance of the intestinal microflora in preserving the organism’s normal functioning has become increasingly acknowledged in recent years. As a result, numerous studies have investigated the potential therapeutic effects of microorganisms found in the intestinal microbiome in a myriad of diseases [134]. In most cases, it is unclear whether changes in the microbiota are a cause or consequence of the disease and whether the manipulation of the gut microbiota can help control or even treat the pathological condition [133]. Some lifestyle changes via alternative treatments, including fecal microbiota transplantation (FMT), diet changes, and dietary supplementation with probiotics, prebiotics, and symbiotics, have indicated possible protective effects in microbiota dysbiosis and promotion of healthy microbes, along with minimal long-term unfavorable effects [135] (Figure 2). Nevertheless, existing microbial-targeted treatments, including probiotics, prebiotics, FMT, and specific dietary regimens, yield mixed results that could be more optimal for everyday clinical use [136,137,138].

### 6.1. Probiotics

Probiotics are defined as live microorganisms that confer health benefits on the host when administered in appropriate amounts [133,139]. Lactobacilli, streptococci, and bifidobacteria are human food’s most common probiotic bacteria. Furthermore, the yeast *Saccharomyces boulardii* and a strain of *E. coli* known as the Nissle strain are frequently used [133]. Probiotics stimulate the production of anti-inflammatory cytokines and the secretion of antimicrobial substances, suppress the growth of bacteria, induce an immune response, have an immunomodulating role, and improve the epithelial barrier function [134].

Various studies have shown that distinct probiotic strains or a mixture of strains can be rather helpful in various diseases [133,140]. The use of antibiotics can have long-term consequences on the composition of the intestinal microbiota and, thus, on overall health [133,141]. Conversely, research with probiotics showed fewer distortions of the intestinal microbiota when probiotics were given together with antibiotics [133,142]. Probiotics mainly exert their action in the small intestine, where the concentration of resident microbiota is low, affecting the microbiota’s diversity and richness during their passage [133]. Endogenous bacteria will outnumber probiotic bacteria in the large intestine, but they can still (directly and/or indirectly) impact health [133]. Probiotics support beneficial microorganisms in the small intestine, improve barrier integrity, and reduce nutrient malabsorption and pathology associated with small bowel disease. Treatment with specific strains of probiotics represents a natural and effective approach to restore barrier integrity and eubiosis of the small intestine, resulting in improved health and a reduction in the incidence and severity of small bowel disease [143]. In addition, probiotics (especially lactic acid bacteria) can protect the small intestine by increasing microbial diversity, regulating the expression of proteins involved in homeostasis, and maintaining the integrity of the immune system [144]. Although bile acids and digestive enzymes can affect the viability of probiotics in the small intestine, they are coated with a protective layer, which increases the survival rate of viable probiotics, and more can reach the large intestine. However, as the large intestine has the highest density of bacteria, probiotics encounter resistance from commensal bacteria; as a result, probiotics must compete with the host microbiota for nutrients and adhesion sites to colonize the colonic mucosa and proliferate. Due to their resistance to colonization, most probiotics are excreted from the colon in the stool after oral administration and soon after consumption, so probiotics are undetectable [145]. Probiotics can also act as prebiotics, promoting the growth of specific intestinal microbiota [133]. Metabolic by-products, dead microorganisms, bacterial molecular components, or other nonviable microbial-based products are not, by definition, probiotics, but exhibit probiotic properties [146]. Probiotics can stimulate the growth of part of the intestinal microbiota. For example, some studies have shown that a probiotic strain of *Lactobacillus casei* increased the concentration of lactobacilli in the stools of young children. Additionally, the *Lactococcus lactis* strain increases the concentration of bifidobacteria and decreases the concentration of *Enterococcus* in human-flora-associated rats [147]. Moreover, the levels of commensal bacteria in the ileum of rats correlated with the positive disease outcome of prophylactic probiotic therapy in a rat model of acute pancreatitis [148]. The success of using probiotics in treating IBD can vary, and primarily depends on the strains used and the target subtype of the disease [149]. Probiotics currently available can potentially modulate dysbiosis in IBD patients, but their effects are temporary [136,138,150]. The most ordinary types of probiotics used are *Bifidobacterium* and *Lactobacillus* [136,150], which have proven to be effective in UC therapy, and are based on the immunomodulatory and anti-inflammatory effects of probiotics [134]. Visbiome is the most common probiotic cocktail with proven efficacy in UC [135,151]. More specifically, Visbiome contains eight different strains of bacteria, and the most frequently used bacteria are *Lactobacilli* and *Bifidobacteria* [135]. Most studies have shown the effectiveness of probiotics in maintaining clinical remission of CD [134]. However, there is insufficient evidence to support probiotics as an adjunctive therapy in CD patients [134,152]. Since the performance of these probiotics is not entirely satisfactory, new candidates for more effective colonizing probiotics—such as combinations of protective resident strains (live biotherapeutic products, LBP)—are emerging [136,153].

### 6.2. Prebiotics

Prebiotics are nondigestible carbohydrates that are metabolized by resident bacteria and can enhance the composition and metabolic function of beneficial resident bacterial species in the intestine [136,154]. A prebiotic can be defined as a selectively fermented ingredient that stimulates specific changes in the activity or composition of the gut microbiota, providing benefits to the host [133]. Health benefits extend beyond the GIT to the skin, urogenital tract, and lungs [135,155]. Prebiotics are carbohydrate polymers that are not absorbed, as opposed to probiotics (which are living microorganisms) [134]. Prebiotics promote the growth and/or activity of bacteria found in the large intestine and act as growth substrates to selectively increase the number of certain bacteria and/or their activity [133]. Commensal microorganisms ferment them, which leads to a change in the composition of the intestinal microbiome and metabolism [135,155].

Prebiotics include nondigestible carbohydrate sources, such as fructooligosaccharides, inulin, and galactooligosaccharides [134,136,154], but also non-carbohydrate sources such as polyphenols and specific lipids [135,155]. All these compounds promote the growth and metabolic activity of beneficial bacteria found in the digestive tract, such as *Lactobacillus* and *Bifidobacterium* species [134]. In addition to the benefits mentioned above, prebiotics stimulate the bacterial production of short-chain fatty acids, such as butyrate, which has immunoregulatory properties. These effects include the suppression of pro-inflammatory cytokines [134,156].

In clinical use, prebiotics may show benefits in treating IBD, but their effects are modest, with conflicting results [136,157]. Thus, *F. prausnitzii* has been shown to respond to prebiotic supplementation using fructans of mixed chain length [133,158], and decreased numbers of *F. prausnitzii* have been observed in patients with CD [133].

### 6.3. Synbiotics

Synbiotics combine selected prebiotics and probiotics to achieve a synergistic effect. Probiotics suppress the development of pathogenic bacteria, while prebiotics stimulate the proliferation of beneficial intestinal microbes and thus synergistically enhance the integrity of the intestinal barrier [135]. The effectiveness of the oral administration of synbiotics is slightly higher than the independent administration of probiotics and prebiotics [134]. Unfortunately, data on the usefulness of synbiotics in patients with IBD are scarce [134,159]. In CD, the use of prebiotics did not lead to a considerable improvement in the patient’s health condition. However, the combined use of prebiotics with *Bifidobacterium longum* in patients with active CD improved clinical symptoms [134,160].

### 6.4. Dietary Modifications

It has been shown that there is a connection between a change in usual diet and a diet with specific ingredients that can be risk factors for IBD [161,162], showing that we can manipulate intestinal microbes by intervening in human nutrition [163]. A simple example is the use of specific fiber sources that provide food for the desired intestinal microbes [154,163]. Intestinal microbes adapt to the new food environment, whereby the bacteria in the human intestines contribute to the body’s response to a particular diet [163]. Thus, it was shown that at least two weeks of consuming vegetables rich in inulin fructans in the diet increased the genus *Bifidobacterium* by 3.8 times [134]. In addition, the intestinal microbiota and intestinal immunity are affected by numerous polyphenols. The large intestine receives a significant amount of unabsorbed polyphenolic compounds, which causes them to interact with the large intestine’s intestinal microbiota, catabolizing, in turn, polyphenols and breaking them down into little fragments [134,164].

Diet has a more significant impact on CD than UC, since the majority of epidemiological research on dietary risk factors has determined an association with CD but not UC [135,165]. Special attention has been paid to the Mediterranean diet in terms of inducing and maintaining CD remission [135]. In addition, diet is an influential lifestyle factor significantly related to the function of the gut microbiota [135]. It also provides valuable insight into developing personalized nutritional strategies to adapt gut microbes in the future [163].

### 6.5. Fecal Microbiota Transplantation (FMT)

FMT involves collecting feces from a healthy donor and transplanting it into the patient’s gastrointestinal tract [134], replacing the patient’s dysbiotic gut microbiota with microorganisms from healthy donors [149]. FMT’s goal is to restore healthy microbiota [135,166], which is nowadays not only possible, but often a preferred therapeutic alternative in patients with a disturbed intestinal ecosystem. This specific therapeutic method is highly successful in patients with recurrent *Clostridioides difficile* infection, particularly with strains resistant to standard antibiotic therapy [134,135,136,167].

By triggering many immune-mediated pathways [134,168], FMT can benefit the restoration of intestinal dysbiosis, the production of pro-inflammatory factors [134,169], the reduction of intestinal inflammation, and the promotion of the restoration of intestinal homeostasis [134]. However, numerous inconsistencies in the results of FMT are a consequence of the complex pathogenesis of IBD in relation to CD [135]. Preliminary studies using FMT in patients with UC or CD showed promising results in many cases in achieving and maintaining long-term clinical remission. However, the benefits were more significant in younger patients [134]. Furthermore, the effectiveness of FMT in the IBD patient is still controversial [136,150,167] because FMT can potentially cause grave and life-threatening side effects [136].

Different mixtures of intestinal protective microbial strains or their metabolites may be safer and more adequate than the entire FMT. Therefore, many new LBP formulations are being designed to replace protective bacterial species in IBD patients [136], and novel solutions are being developed in line with precision medicine postulates.

## 7. Conclusions

The intestinal microbiota comprises various microorganisms, including proteobacteria, bacteria, viruses, fungi, protists, and archaea. The microbiota has a significant role in the GIT, such as participating in the metabolism of various substances and steering the activity of the immune system. Therefore, changes in the composition of the microbiome of the GIT and its function, known as dysbiosis, play a noteworthy role in the development of chronic IDB that entails CD and UC. In order to successfully treat clinical conditions that arise from dysbiosis, it is pivotal to closely investigate the microbiota’s influence and its role in pathogenesis, as well as the currently available therapeutic approaches. In addition, we should be cognizant that adequate probiotic preparations can aid in treating IBD. It is evident that the microbiome is closely linked to a plethora of host phenotypes, so the capability to engineer our commensal microflora will likely be an indispensable element of personalized or precision medicine during this century.

## Figures and Tables

**Table 1:** Types of dysbiosis.

No	Dysbiosis Type	Reference
1	Loss of beneficial bacteria	[78]
2	Overgrowth of potentially pathogenic bacteria	[78]
3	Loss of overall bacterial diversity	[78]

**Table 2:** Diseases for which dysbiosis is a crucial factor.

Disease	Reference
Atherosclerosis	[79]
Autism	[81]
Crohn’s disease	[78,79,81]
Diabetes mellitus type 1 and 2	[79]
Rheumatoid arthritis	[79]
Ulcerative colitis	[78,79,81]

**Table 3:** List of altered bacteria in inflammatory bowel disease.

	Phylum	Genera	Reference
Reduced	Firmicutes	*Bacillus*	[88]
		*Clostridium*	[88]
		*Enterococcus*	[88]
		*Lactobacillus*	[88]
		*Ruminicoccus*	[88]
Increased	Bacteroidetes	*Alistipes*	[88]
		*Bacteroides*	[88]
		*Parabacteroides*	[88]
		*Prevotella*	[88]
	Proteobacteria	*Escherichia coli*	[89]
		*Klebsiella*	[89]
		*Shigella*	[89]

## Data Availability

Not applicable.
